# Treatment rates and barriers to mental health service utilisation among university students in South Africa

**DOI:** 10.1186/s13033-023-00605-7

**Published:** 2023-11-09

**Authors:** Jason Bantjes, Molly J. Kessler, Xanthe Hunt, Dan J. Stein, Ronald C. Kessler

**Affiliations:** 1 Mental Health, Alcohol, Substance Use and Tobacco (MAST) Research Unit, South African Medical Research Council, Cape Town, South Africa; 2 Department of Psychiatry and Mental Health, University of Cape Town, Cape Town, South Africa; 3 Boston College, Chestnut Hill, MA USA; 4 Institute for Life Course Health Research, Department of Global Health, Stellenbosch University, Stellenbosch, South Africa; 5 SAMRC Unit on Risk and Resilience in Mental Disorders, Department of Psychiatry, Stellenbosch University, Stellenbosch, South Africa; 6 Department of Health Care Policy, Harvard Medical School, Boston, MA USA

**Keywords:** University students, Mental health problems, Mental disorders, Suicidality, Treatment rates, Treatment barriers

## Abstract

**Background:**

Mental health problems are common and impairing among university students, yet only a minority of students with psychological disorders access treatment. Understanding barriers to treatment is integral to planning services, especially in resource constrained settings like South Africa (SA).

**Methods:**

Data collected across 17 institutions in the online *SA National Student Mental Health Survey* were used to: (1) estimate 12-month prevalence of common mental health problems and self-harm; (2) estimate the proportion of students receiving treatments for the various mental health problems; (3) explore barriers to treatment; and (4) investigate sociodemographic predictors of treatment mediated through the various barriers endorsed by students with mental health problems. Prevalence analyses were carried out using cross-tabulations and prediction analyses using modified Poisson regression models.

**Results:**

Prevalence of clinically significant mental health problems is high relative to international comparisons, with the prevalence of severe, mild and moderate symptoms of any disorder and/or self-harm of 24.8% (SD = 0.3), 18.8% (SD = 0.3) and 27.6% (SD = 0.4) respectively. Treatment rates were 35.2% (S.E. = 0.6) among students with mental health problems who perceived need for treatment and 21.3% (S.E. = 0.4) irrespective of perceived need. Treatment rates were highest for mood disorders (29.9%, S.E. = 0.6) and lowest for externalising disorders (23.8%, S.E. = 0.5). Treatment rates were much less variable across disorder types among students with perceived need than irrespective of perceived need, indicating that perceived need mediated the associations of disorder types with received treatment. Adjusting for disorder profile, probability of obtaining treatment was significantly and positively associated with older age, female gender, study beyond the first year, traditional sexual orientation, and diverse indicators of social advantage (full-time study, high parent education, and attending Historically White Institutions). Among students with mental health problems, numerous barriers to treatment were reported adjusting for disorder profile, including lack of perceived need (39.5%, S.E. = 0.5) and, conditional on perceived need, psychological (54.4%, S.E. = 1.0), practical (77.3%, S.E. = 1.1), and other (79.1%, S.E. = 1.1) barriers. Typically, students reported multiple barriers to treatment. Differences in perceived need explained the gender difference in treatment, whereas practical barriers were most important in accounting for the other predictors of treatment.

**Conclusion:**

Mental health problems are highly prevalent but seldom treated among SA university students. Although many barriers were reported, practical barriers were especially important in accounting for the associations of social disadvantage with low rates of treatment. Many of these practical barriers are however addressable.

**Supplementary Information:**

The online version contains supplementary material available at 10.1186/s13033-023-00605-7.

## Introduction

Mental disorders and self-harm are common among university students both globally [1, 2] and in South Africa (SA) [3–5]. An international survey of first-year students (n = 13,984) from 19 universities across 8 countries reported a 31.4% 12-month prevalence of any common mental disorder [6], and a 17.2% 12-month prevalence of suicidality [7]. Our large national survey of students (*n* = 28,268) from 17 SA universities reported 30-day prevalence estimates of 16.3% for mood disorders, 37.1% for anxiety disorders [4] and 24.4% for suicidal ideation [8]^.^ Left untreated, these conditions can impede functioning [9] and lead to academic failure [10, 11] and suicide [12]. Although evidence-based treatments are available [13], previous studies have established that typically only a minority of students with mental disorders receive treatment [14].

Although considerable research has been carried out on treatment barriers for mental disorders among university students, most studies have been conducted in the USA and very few in low- and middle-income countries [15]. The exception in SA was a study of first-year students at two well-resourced universities (n = 1402), which found 28.9–35.0% treatment rates among first-year students with mental disorders and suicidality [16], but did not investigate barriers to treatment. Understanding such barriers would be important for planning campus-based services. Research in other countries suggests that availability, accessibility, affordability, and a range of institutional (i.e., contextual and structural) and individual (i.e., perceived need for treatment and attitudinal) factors influence utilization of mental health care [17].

Understanding and overcoming barriers to treatment seeking is integral to a public mental health approach to student wellness, particularly in resource-constrained environments like SA, where services need to be organized to maximize the benefits of scarce mental health resources [18]. To date, no studies have systematically investigated treatment rates for mental health problems and barriers to accessing treatment among SA university students. At a national level it is also important to explore measures of social disadvantage, particularly disparities across the four main types of SA universities: (1) Historically White Institutions (HWIs), which prior to 1994 were predominantly reserved for the country’s “White” population and still are typically better resourced then other institutions; (2) Historically Disadvantaged Institutions (HDIs) established for Black students and mostly located in rural settings and former homelands (i.e., geographic regions set aside by the Apartheid Government for Black inhabitants to keep them from living in the urban areas reserved for “Whites”) [19]; (3) “Universities of Technology” (UTs), primarily focused on vocational education and developing students’ capabilities to use technology [20]; and (4) a single large distance learning university (DLU), with an enrolment of approximately 370,000, that serves many part-time and older students. In this context, it is important to note that the use of terms like “White” and “Black” to describe particular population groups is an artifact of the country’s political history of apartheid, and that these terms continue to be used in official documents and for population census purposes. The term “White” typically refers to individuals who are considered to be of European descent, while the term “Black” denotes those with ancestry other than European (including African, Colored, Asian and Indian). Our use of these terms here is not intended to imply any biological basis for these categories nor is it intended to essentialize notions of race. We have used these terms as a way to investigate ongoing social disparities and inequalities in access to education and other resources as a result of the country’s political history of segregation.

## Methods

Data collected as part of the *SA National Student Mental Health Survey* were used to: (1) estimate 12-month prevalence of common mental health problems and self-harm; (2 estimate the proportion of students receiving treatments for the various mental health problems; (3) explore barriers to treatment; and (4) investigate sociodemographic predictors of treatment as mediated through barriers. The study is part of the ongoing work of the *World Health Organization* (WHO) *World Mental Health Surveys International College Student Initiative* (WMH-ICS) [21], which seeks to expand access to evidence-based treatments for mental disorders among students across the globe.

### Procedure

All 26 public universities in SA were invited to participate in the survey, of which 17 agreed to be included. No reasons were given by the 9 non-participating universities, among which there were 2 HWIs, 3 HDIs and 4 UTs. Data were collected between April and October 2020. Participating universities distributed emails inviting all their undergraduate students to complete an anonymous online survey (N = 657,432). Two follow-up reminder invitations were sent to students, approximately a week apart. The study was initiated by *Universities South Africa* and funded by *the South African Medical Research Council*.

### Procedures and measures

The survey was administered via Qualtrics (a web-based platform used for administering electronic surveys). Responses were self-administered by participating students and the following information was obtained:

#### Socio-demographic characteristics

Students reported their age, gender, population group, sexual orientation, parents’ education and whether they were full-time or part-time students. For population group we used the categories in government policies and the official population census (i.e., Black-African, Coloured, White, Asian, and “Other”) to explore disparities in mental health utilisation that may have resulted from the country’s history of racial segregation.

#### Mental health problems

Self-report information was collected to assess 11 common mental health problems, including 4 anxiety-based disorders (generalized, anxiety disorder (GAD), panic disorder, post-traumatic stress disorder (PTSD), social phobia), 2 mood disorders (major depressive episode (MDE), bipolar spectrum disorder), 3 disruptive behavior disorders (ADHD, eating disorder, intermittent explosive disorder) and 2 substance use disorders (alcohol use disorder, drug use disorder). We used the *Composite International Diagnostic Interview Screening Scales* (CIDI‐SC) [22, 23] to assess all disorders other than for alcohol use disorder, which we assessed using the *Alcohol Use Disorders Identification Test* (AUDIT) [24]. Previous cross-national research has documented good validity of these assessments compared to clinical evaluations [22, 23, 25].

#### Self-harm

Suicidal thoughts and behaviours were assessed using a modified version of the *Columbia Suicidal Severity Rating Scale* (C-SSRS), which has demonstrated good convergent and divergent validity with other multi-informant suicidal ideation and behavior scales used with adolescents, as well as showing high sensitivity and specificity for suicidal behavior classifications compared with other behavior scales and clinician evaluation [26]^.^ Students were asked about passive suicidal ideation (i.e*. wish you were dead or would go to sleep and never wake up*), active suicidal ideation (i.e*. thoughts of killing yourself),* suicide plans* (*i.e*. think about how you might kill yourself),* suicide attempts* (*i.e*. purposefully hurt yourself with at least some intent to die), and* non-suicidal self-injury (NSSI) (i.e*. do something to hurt yourself on purpose, without wanting to die, like cutting yourself, hitting yourself, or burning yourself)*). Students who endorsed any of these items where then asked which of these problems occurred within the past 12-months.

#### Symptom severity

To measure level of impairment related to mental health problems (i.e. severity of symptoms) we used the *Mental Component Score (MCS)* of the *Veterans RAND 12-Item Health Survey* (VR-12) [27]. The VR-12 is a 12-item scale assessing 8 domains of health; namely, physical functioning, role limitation due to physical problems, bodily pain, general perception of health, social functioning, role impairment due to emotional problems, vitality, and mental health. The MCS was derived from the VR-12 questions assessing social functioning, role limitation due to emotional problems, vitality, and mental health. These items were then rescaled to yield a score ranging from 0 to 100, with higher scores indicating better health and less impairment [28]. The MCS has a mean of 50 and SD of 10 in the US population. Students who scored two standard deviations (SD) below the mean were defined as having severe symptoms while those who scored between one and two SDs below the mean were defined as having moderate symptoms and those who scored less than 1 SD below the mean were defined as having only mild symptoms.

#### Mental healthcare utilization

Students were asked if they had ever accessed treatment for an emotional or substance use problem and, if so, whether this occurred in the preceding 12-months. If treatment was received, the assessment asked separately if the treatment included psychological counselling, medication, or both.

#### Perceived need for treatment

We assessed perceived need for treatment by asking students who did not obtain treatment: *Was there ever a time in the past 12-months when you felt that you might need psychological counseling, medication, or some other type of treatment for any emotional or substance use problems?* Only students who answered affirmatively were queried about barriers to treatment.

#### Barriers to treatment utilisation

Students who did not receive treatment even though they screened positive for one or more of the 11 common mental health problems we assessed and/or self-harm and recognized a need for treatment were then asked about the importance of 9 barriers to treatment seeking commonly reported in prior student surveys (see footnote to Table 2).

### Data processing

Standard calibration methods were used to weight the data within institutions to adjust for differences between survey respondents and the population on profiles defined by gender, population group, and year in school [29]. A second weight was then used to adjust for differences in survey response rates *between* institutions (Additional file 1: Table S1). Full descriptions of weighting procedures are reported elsewhere [4]. Multiple imputation (MI) across 30 MI replicates by chained equations was used to adjust for item-missing data [30].

### Data analysis

We calculated 12-month prevalence estimates for mental health problems and self-harm, as well as gross associations with perceived need and treatment with cross-tabulations across the 30 multiple imputed datasets using Rubin’s rule [31]. MI-adjusted standard errors to adjust for the weighting and clustering of observations were obtained through the Taylor series linearization method [32]. We then used a data-driven method, random forests (RF) regression [33], to estimate the joint associations of the various groups of mental health problems (i.e. anxiety disorders, mood disorders, externalizing disorders, and self-harm) with probability of obtaining treatment. Given the computational complexity of RF using MI, the RF analysis was carried out at the person level among respondents who were imputed to have at least one condition in at least one imputation using counts of number of imputed with each condition imputed to be present. We retained the individual-level predicted probability of treatment based on this RF analysis as a control variable in subsequent prediction analyses described below.

Before carrying out other prediction analyses, though, we assessed the structure of reported barriers to treatment using principal axis factor analysis with oblique rotation to investigate the structure among responses to the questions about barriers (see the footnote to Table 2 for a full description of the 9 barriers). Missing values were imputed to the mean in carrying out this analysis. We then created summary dichotomous measures to describe whether each student reported one or more barriers within each factor to be either a *very important* or an *important* reason for not obtaining treatment. We generated a Venn diagram to examine the inter-correlations among these reports to define multivariable barrier profiles.

The prediction analyses used Poisson regression models with robust error variances [34] to estimate associations of sociodemographic factors and university type (i.e., HWI, HDI UT, DLU) with perceived need, barriers among students with perceived need, treatment, and treatment controlling for the RF predicted probability associated with disorder profiles. Poisson regression coefficients and ± two standard errors of these coefficients were exponentiated to create risk ratios (RRs) and 95% confidence intervals (95% CI).

We then decomposed the significant RRs of sociodemographic factors and university type with treatment by re-estimating the Poisson regression model in subsamples that excluded students with no perceived need and then successively excluded students with each type of barrier. This subsample analysis was used rather than control variable analysis (i.e., controlling for perceived need and barriers in a multivariable model) because control variable analysis is not possible when none of the people with the control variables received treatment. The importance of perceived need and barriers in explaining the RRs of the predictors with treatment was inferred in the subsample analysis by examining changes in RRs when we excluded respondents who lacked perceived need or reported various barriers.

### Ethics

Ethical clearance was provided by the Health Science Research Ethics Committee of Stellenbosch University (Reference: N13/10/149). Institutional permission was obtained from all participating universities. Students provided informed consent electronically prior to data collection. Information about crisis and student counselling services was provided to all participants. Anonymised and de-identified data were securely stored on a password protected cloud-based server. The research was performed in accordance with the Declaration of Helsinki.

## Results

### Sample characteristics

28,516 students completed the survey. Twelve-month prevalence of any assessed mental health problem (i.e., disorders and self-harm) was 71.3% (S.E. = 0.5) (Table 1), with anxiety disorders the most common group (53.6%, S.E. = 0.4) and social anxiety the most common anxiety disorder (37.2%, S.E. = 0.4). Of all students, 45.1% (S.E. = 0.4) reported an externalising disorder, with binge eating the most common (22.2%, S.E. = 0.3). Self-harm (39.7%, S.E. = 0.4) and mood disorders (29.9%, S.E. = 0.2) were least common. In the total sample the prevalence of severe, mild and moderate symptoms of any disorder and/or self-harm were 24.8% (SD = 0.3), 18.8% (SD = 0.3) and 27.6% (SD = 0.4) respectively (Table 1). This means that roughly one-third of all students with a mental health problem had a severe problem (i.e., 24.8%/71.3%), another one-fourth a moderate problem (i.e., 18.8%/71.3%), and the final roughly 40% a mild problem (i.e., 27.6%/71.3%).

**Table 1:** Prevalence of 12-month disorders, perception of need for treatment and treatment rates

	Prevalence of disorder			Perceived need for treatment among students with the disorder			Treatment among students who perceive a need for treatment			Treatment among students with the disorder		
	N	%	S.E	N	%	S.E	N	%	S.E	N	%	S.E
GAD	5302	18.8	0.2	4397	82.9	1.1	1788	40.7	1.0	1788	33.7	0.8
Panic disorder	2790	9.9	1.0	2294	82.2	8.6	1021	44.5	4.7	1021	36.6	3.9
Social anxiety disorder	10,502	37.2	0.4	7224	68.8	0.8	2488	34.4	0.8	2488	23.7	0.5
PTSD	10,183	36.0	0.4	7602	74.7	0.9	2776	36.5	0.8	2776	27.3	0.6
**Any anxiety disorder**	**15,151**	**53.6**	**0.4**	**10,233**	**67.5**	**0.7**	**3627**	**35.4**	**0.7**	**3627**	**23.9**	**0.5**
Bipolar disorder	740	2.6	0.2	601	81.2	5.9	265	44.1	4.0	265	35.8	3.2
MDE	8226	29.1	0.2	6703	81.5	0.9	2470	36.8	0.8	2470	30.0	0.6
**Any mood disorder**	**8461**	**29.9**	**0.2**	**6857**	**81.0**	**0.9**	**2526**	**36.8**	**0.8**	**2526**	**29.9**	**0.6**
ADHD	5597	19.8	0.3	4259	76.1	1.1	1605	37.7	1.0	1605	28.7	0.7
Binge eating	6275	22.2	0.3	4176	66.6	1.2	1490	35.7	1.1	1490	23.7	0.7
Purging	1973	7.0	0.2	1128	57.2	2.2	440	39.0	2.4	440	22.3	1.4
Alcohol use disorder	1893	6.7	0.2	1325	70.0	2.9	494	37.3	2.6	494	26.1	1.8
Drug use disorder	2966	10.5	0.2	2188	73.8	2.0	932	42.6	1.8	932	31.4	1.3
**Any externalizing disorder**	**12,760**	**45.1**	**0.4**	**8315**	**65.2**	**0.8**	**3042**	**36.6**	**0.8**	**3042**	**23.8**	**0.5**
Suicide ideation	10,958	38.8	0.4	7926	72.3	0.9	2886	36.4	0.8	2886	26.3	0.6
Suicide plan	5181	18.3	0.4	4164	80.4	1.7	1580	37.9	1.3	1580	30.5	1.1
Suicide attempt	1282	4.5	0.2	1126	87.8	3.7	487	43.3	2.8	487	38.0	2.4
Non-suicidal self-injury (NSSI)	2266	8.0	0.2	1945	85.8	2.5	850	43.7	2.0	850	37.5	1.7
**Any self-harm**	**11,233**	**39.7**	**0.4**	**8103**	**72.1**	**0.9**	**2955**	**36.5**	**0.8**	**2955**	**26.3**	**0.6**
Severe symptoms of any disorder and/or self-harm	7024	24.8	0.3	5782	82.3	1.1	2061	35.6	0.9	2061	29.3	0.7
Moderate symptoms of any disorder and/or self-harm	5319	18.8	0.3	3295	62.9	1.0	1130	34.3	1.2	1130	21.24	0.8
Mild symptoms of any disorder and/or self-harm	7809	27.6	0.4	3122	39.9	1.1	1106	35.42	1.5	1106	14.2	0.6
**Any common mental disorder and/or self-harm**	**20,152**	**71.3**	**0.5**	**12,199**	**60.5**	**0.5**	**4299**	**35.2**	**0.6**	**4299**	**21.3**	**0.4**

Bold text indiciates sub-heading for agregate of any of the disorders in the preceeding rows

### Perceived need for treatment and treatment rates

60.5% of students with any of the mental health problems assessed perceived themselves as needing treatment, and perceived need for treatment was higher amongst students with mood disorders (81.0%, S.E. = 0.9) followed by self-harm (72.1%, S.E. = 0.9), but lower among students with anxiety disorders (67.5%, S.E. = 0.7) and externalising disorders (65.2%, S.E. = 0.8). Perceived need for treatment also varied substantially depending on whether the problems were severe (82.3% [SD = 1.1]), moderate (62.9% [SD = 1.0]), or mild (39.9% [SD = 1.1]).

Treatment rates were 35.2% (S.E. = 0.6) among students with disorders who had perceived need and 21.3% (S.E. = 0.4) irrespective of perceived need. Treatment rates were highest for mood disorders (29.9%, S.E. = 0.6) and lowest for externalising disorders (23.8%, S.E. = 0.5), It is noteworthy that treatment rates were much less variable across disorder types among students with perceived need than irrespective of perceived need, indicating that perceived need mediated the associations of disorder types with received treatment. Strikingly, treatment rates among students who perceived a need for treatment were not related to problem severity, with treatment rates among those whose problems were severe, moderate, or mild of 35.6% (SD = 0.9), 34.3% (SD = 1.2) and 35.4% (SD = 1.5), respectively. This indicates that severity is important for help-seeking largely in leading to perceived need.

### Barriers to treatment

We assessed nine barriers to treatment (see footnote in Table 2 for a full description) and using exploratory factor analysis found two strong factors; namely psychological/attitudinal barriers (too embarrassed; afraid might adversely affect school or professional career; worried people would treat them differently) and practical barriers (unsure where to go; too expensive; problems with time, transportation, or scheduling) (Additional file 1: Table S2). The remaining barriers were unrelated (want to handle problems on their own, preference to talk to family/friends; unsure of treatment effectiveness).

**Table 2:** Barriers to treatment among students with any of the conditions we assessed who perceived a need for treatment but who did not access treatment, by institution

		Total (n = 7912)			HWI (n = 2195)			HDI (n = 779)			UTs (n = 273)			DLU (n = 4653)			F(3)	P
		N	%	S.E	N	%	S.E	N	%	S.E	N	%	S.E	N	%	S.E		
Psychological/attitudinal barriers	Too embarrassed	3029	38.3	0.8	802	36.4	1.3	300	38.5	2.0	110	40.2	3.1	1818	39.0	1.1	9.371	
	Discrimination (social)	2591	32.7	0.7	675	30.7	1.2	251	32.3	1.9	105	38.5	3.2	1559	33.5	1.0		
	Discrimination (career)	2331	29.5	0.7	578	26.3	1.2	214	27.4	1.8	95	34.8	3.1	1444	31.0	1.0		
	Any psychological/attitudinal barrier	4294	54.3	0.9	1149	52.2	1.4	417	53.5	2.1	164	60.2	3.3	2564	55.0	1.3		
Practical barriers	Costs too much money	4488	56.7	0.9	1120	50.9	1.4	432	55.4	2.2	144	52.6	3.2	2792	59.9	1.3	23.238	**<** 0.001*
	Unsure where to go	4621	58.4	0.9	1145	52.0	1.4	460	59.1	2.2	164	60.1	3.3	2852	61.2	1.3		
	Time/scheduling problem	3237	40.9	0.8	837	38.0	1.3	332	42.6	2.1	120	44.1	3.3	1947	41.8	1.1		
	Any Practical barrier	6103	77.1	0.9	1625	73.8	1.4	601	77.2	2.1	204	74.8	3.1	3673	78.8	1.4		
Other barriers	Talk to family/friends instead	2884	36.5	0.8	731	33.2	1.2	267	34.3	1.9	89	32.4	3.1	1797	38.6	1.1	3.247	0.5
	Want to handle on own	4844	61.2	0.9	1385	62.9	1.5	487	62.5	2.3	180	65.8	3.3	2792	59.9	1.3		
	Unsure of effectiveness	2276	28.8	0.7	566	25.7	1.1	230	29.6	1.8	76	27.8	2.9	1403	30.1	1.0		
	Any other barrier	6260	79.1	0.9	1727	78.5	1.5	621	79.8	2.1	226	82.9	2.9	3685	79.1	1.4		
Any important barrier	7509	94.9	0.9	2090	95	1.4	737	95	1.9	257	94	2.4	4425	95	1.4	0.725	0.91	

We assessed barriers to treatment by asking students to rate the importance of various reasons for NOT accessing treatment on a five-point Likert scale (i.e*., How important were each of the following reasons for why you did NOT seek help for your problem(s) – very important, important, somewhat important, not very important, or unimportant?*). The reasons given were: You were too embarrassed; You worried that people would treat you differently if they knew you were in treatment); You were afraid it might harm your school or professional career; It costs too much money; You were unsure of where to go or who to see; You had problems with time, transportation, or scheduling; You talked to friends or relatives instead; You wanted to handle the problem on your own; You were not sure if available treatments were very effective

*P < 0/05

Table 2 shows the proportion of students with perceived need who reported each barrier as being either *very important* or *important*. 54.3% (S.E = 0.9) of the students with disorders who did not receive treatment reported psychological/attitudinal barriers, 77.1% (S.E = 0.9) practical barriers, and 79.1% (S.E. = 0.9) one or more “other” barriers. Significant fewer students in HWIs than other types of institutions reported practical barriers (F = 23.24, p < 0.001), but no significant differences were found across types of institution for other barriers.

**Fig. 1:**
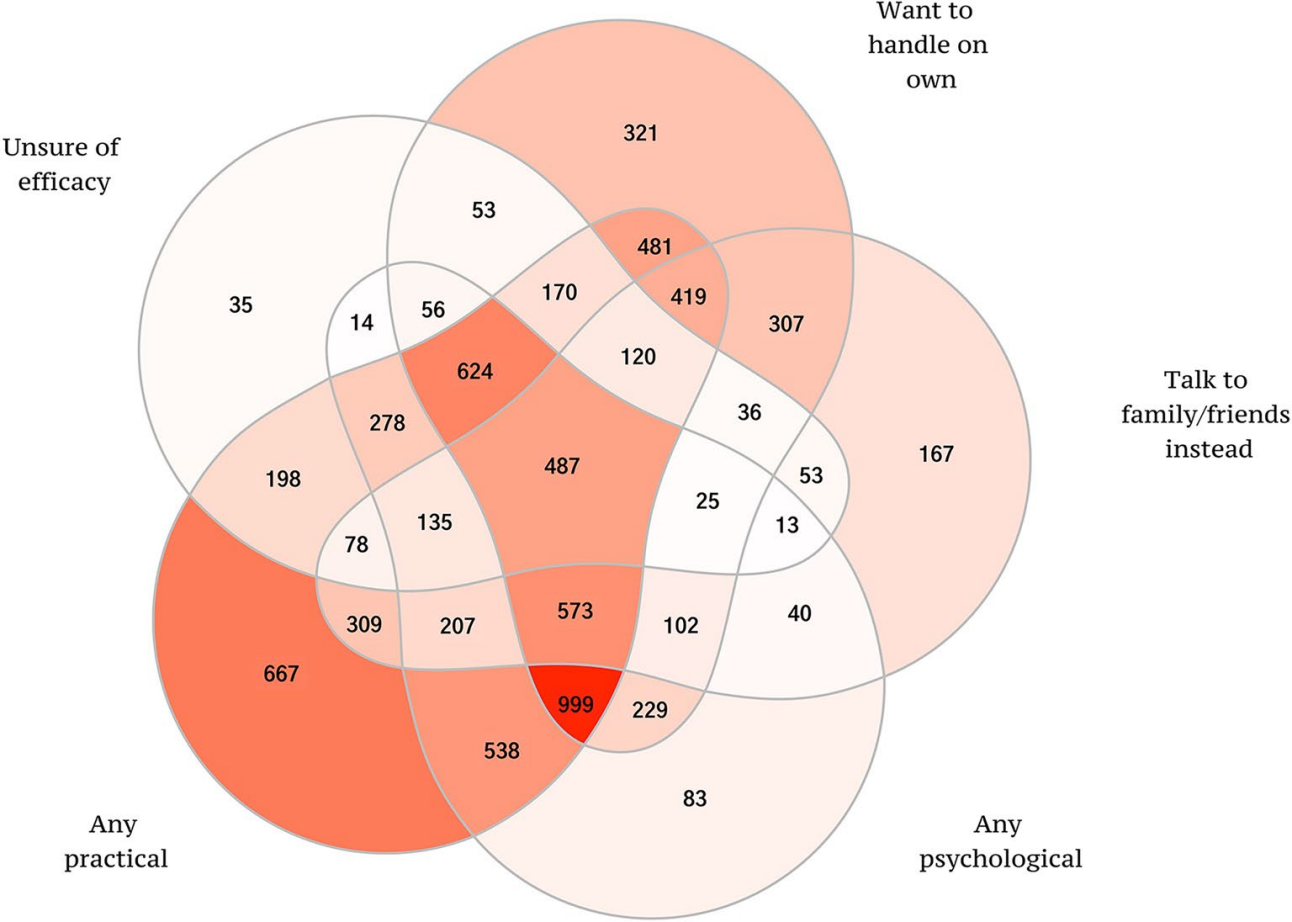
Venn diagram showing the profiles of different barriers to treatment reported by students

The sum of the proportions of students who reported the various barriers was 258%, indicating that the typical student reported multiple barriers. A sense of the most common profiles is provided in the Venn diagram in Fig. 1 (also see Additional file 1: Table S2). About one-sixth (16.3%) of students cited just one type and another 28.5% two types of barriers, with practical barriers by far the most single type (8.5% of all respondents reporting barriers) followed by wanting to handle the problem on their own (4.1%) and the most common two-barrier profiles consisting of practical barriers either with psychological barriers (6.9%) or wanting to handle the problem on their own (6.2%). Profiles involving 3 or more types of barrier were more common, with the most common profiles consisting of (i) a combination of practical and psychological barriers along with wanting to handle the problem on their own (12.8%), (ii) all barriers other than talking to family/friends (8.0%), and (iii) all other barriers than unsure of treatment effectiveness (7.3%).

### Sociodemographic predictors of perceived need and barriers

Table 3 shows multivariate Poisson regression models of socio-demographic predictors of perceived need and barriers. Perceived need was highest for students 20–22 years-old (RR = 1.10, 95% CI 1.05, 1.15), female (RR = 1.29, 95% CI 1.25–1.34) and gender non-conforming (RR = 1.32, 95% CI 1.19–1.46), students, full-time students (RR = 1,07, 95% CI 1,03–1,11), students not in their first year of university (RR = 1.06, 95% CI 1.03–1.10), and sexual minorities (RR = 1.21, 95% CI 1.17–1.25), while being lower for students at DLU (RR = 0.82, 95% CI 0.78–0.85), HDIs (RR = 0.81, 95% CI 0.77–0.85) and UTs (RR = 0.88, 95% CI 0.82–0.95).

**Table 3:** Socio-demographic predictors of barriers among respondents with any 12-month mental disorder and perceived need (multivariable Poisson regression models with dichotomous outcome)

	Perceived need for treatment		Any psychological/attitudinal barrier		Any practical barrier		Prefer to talk to family/friends		Want to handle on own		Unsure of effectiveness of treatments	
	IRR	95% CI	IRR	95% CI	IRR	95% CI	IRR	95% CI	IRR	95% CI	IRR	95% CI
**Age X**^2^(4) p	**63.97**	***p*** **<** 0.001*	**46.2**	***p*** **<** 0.001*	**51.61**	***p*** **<** 0.001*	**6.53**	***p*** **=** **0.16**	**42.68**	***p*** **=** **0.16**	**5.35**	***p*** **=** **0.25**
17–19	–	–	–	–	–	–	–	–	–	–	–	–
20–22	***1.10***	***1.05, 1.15***	0.99	0.90, 1.09	0.94	0.88, 1.01	0.9	0.79, 1.03	0.9	0.79, 1.03	1.12	0.96, 1.31
23–25	***1.11***	***1.06, 1.17***	0.91	0.82, 1.02	0.91	0.84, 0.99	0.97	0.84, 1.12	0.97	0.84, 1.12	1.16	0.97, 1.38
26–30	1.03	0.97, 1.09	***0.88***	***0.78, 0.98***	***0.84***	***0.77, 0.92***	1.02	0.87, 1.19	1.02	0.87, 1.19	***1.22***	***1.01, 1.47***
31	***0.90***	***0.84, 0.96***	***0.70***	***0.61, 0.80***	***0.71***	***0.64, 0.78***	0.93	0.78, 1.10	0.93	0.78, 1.10	1.13	0.91, 1.39
**Gender** X^2^(2) p	**171.1**	***p*** **<** 0.001*	**9.88**	***p*** **=** 0.007*	**0.36**	***p*** **=** **0.83**	**4.92**	***p*** **=** **0.09**	**31.99**	***p*** **=** **0.09**	**7.24**	***p*** **=** 0.027*
Male	–	–	–	–	–	–	–	–	–	–	–	–
Female	***1.29***	***1.25, 1.34***	***0.92***	***0.85, 0.98***	1	0.95, 1.05	0.98	0.89, 1.08	0.98	0.89, 1.08	0.96	0.86, 1.07
Gender non-conforming	***1.32***	***1.19, 1.46***	***0.62***	***0.40, 0.98***	0.9	0.67, 1.20	0.48	0.23, 0.97	***0.48***	***0.23, 0.97***	***0.34***	***0.13, 0.89***
**Population group** X2(2) p	**3.6**	***p*** **=** **0.17**	**74.4**	***p*** **<** 0.001*	**112.6**	***p*** **<** 0.001*	**52.82**	***p*** **<** 0.001*	**68.01**	***p*** **<** 0.001*	**130.5**	***p*** **<** 0.001*
White	–	–	–	–	–	–	–	–	–	–	–	–
Black—other	0.95	0.90, 1.00	***1.44***	***1.26, 1.65***	***1.55***	***1.40, 1.71***	***1.39***	***1.17, 1.65***	***1.39***	***1.17, 1.65***	***1.65***	***1.29, 2.10***
Black-African	0.95	0.91, 0.99	***1.6***	***1.44, 1.79***	***1.62***	***1.49, 1.77***	***1.59***	***1.39, 1.82***	***1.59***	***1.39, 1.82***	***2.44***	***2.01, 2.96***
**Student Status** X^2^(1) p	**8.25**	***p*** **=** 0.004*	**0.89**	***p*** **=** **0.35**	**0.193**	***p*** **=** **0.66**	**0.965**	***p*** **=** **0.33**	**0.136**	***p*** **=** **0.33**	**0.96,**	***p*** **=** **0.33**
Full-time degree	–	–	–	–	–	–	–	–	–	–	–	–
Part-time degree	***0.93***	***0.90, 0.97***	0.96	0.89, 1.05	0.99	0.93, 1.04	1.05	0.94, 1.17	1.05	0.94, 1.17	0.95	0.84, 1.08
**Year in school** X^2^(1) p	**6.12**	***p*** **=** 0.013*	**5.93**	***p*** **=** 0.015*	**3.067**	***p*** **=** **0.08**	**0.394**	***p*** **=** **0.53**	0.136,	***p*** **=** 0.194,	0.96	***p*** **=** **0.06**
1st year	–	–	–	–	–	–	–	–	–	–	–	–
All other	***1.06***	***1.03, 1.10***	***0.9***	***0.85, 0.96***	***0.94***	***0.90, 0.98***	1.03	0.95, 1.12	1.03	0.95, 1.12	***0.9***	***0.82, 0.98***
**Parent Education** X^2^(4) p	**2.94**	***p*** **=** **0.40**	**55.4**	***p*** **<** 0.001*	**44.02**	***p*** **<** 0.001*	**14.93**	***p*** **=** 0.002*	**9.798,**	***p*** **=** 0.002*	**31.46**	***p*** **<** 0.001*
University Graduate	–	–	–	–	–	–	–	–	–	–	–	–
Less than secondary	0.97	0.92, 1.02	***1.36***	***1.24, 1.50***	***1.19***	***1.11, 1.28***	1.11	0.97, 1.26	1.11	0.97, 1.26	***1.37***	***1.18, 1.58***
Secondary Graduate	0.98	0.94, 1.02	***1.28***	***1.18, 1.39***	***1.23***	***1.16, 1.31***	***1.2***	***1.08, 1.33***	***1.2***	***1.08, 1.33***	***1.3***	***1.15, 1.48***
Some post-secondary education	1.01	0.97, 1.05	***1.14***	***1.04, 1.24***	***1.17***	***1.10, 1.25***	1.1	0.98, 1.23	***1.1***	***0.98, 1.23***	***1.21***	***1.06, 1.39***
**Sexual orientation** X^2^(1) p	**66.32**	***p*** **<** 0.001*	**0.87**	***p*** **=** **0.35**	**5.703**	***p*** **=** 0.017*	**12.7**	***p*** **<** 0.001*	**14.656**	***p*** **<** 0.001*	**0**	***p*** **=** **0.99**
Heterosexual	–	–	–	–	–	–	–	–	–	–	–	–
All other	***1.21***	***1.17, 1.25***	0.96	0.89, 1.04	***0.92***	***0.87, 0.98***	***0.84***	***0.75, 0.94***	***0.84***	***0.75, 0.94***	1	0.89, 1.13
**Institution type** X^2^(4) p	**67.79**	***p*** **<** 0.001*	**24.3**	***p*** **<** 0.001*	**35.78**	***p*** **<** 0.001*	**5.221**	***p*** **=** **0.16**	**19.388,**	***p*** **=** **0.16**	**5.45**	***p*** **=** **0.14**
HWI	–	–	–	–	–	–	–	–	–	–	–	–
DLU	***0.82***	***0.78, 0.85***	***1.24***	***1.13, 1.35***	***1.26***	***1.18, 1.34***	1.12	1.00, 1.27	***1.12***	***1.00, 1.27***	1.11	0.96, 1.29
HDI	***0.81***	***0.77, 0.85***	***1.13***	***1.02, 1.25***	***1.17***	***1.09, 1.26***	1.12	0.98, 1.29	1.12	0.98, 1.29	***1.19***	***1.01, 1.39***
UT	***0.88***	***0.82, 0.95***	***1.3***	***1.14, 1.48***	***1.17***	***1.06, 1.30***	1.03	0.83, 1.26	1.03	0.83, 1.26	1.08	0.85, 1.35

The term “White” refers to individuals who self-report that they are of European descent, while the term “Black-African” refers to those who self-report that they are of African descent and the term “Black-other” refers to those who self-identify being of ancestry other than European or Black-African

Bold text indiciates a significant association (i.e *p* < 0.05)

Bolditalics text indiciates significant associations

*P < 0/05

Table 3 also shows the multivariate Poisson regression models predicting barriers to treatment among students with perceived need for treatment. Age was inversely related to both psychological/attitudinal and practical barriers, but positively related to being unsure of treatment effectiveness. Males were more likely than others to report psychological/attitudinal and practical barriers, whereas gender nonconforming students were much less likely than others to want to handle the problem on their own or doubt treatment effectiveness. Students identifying as Black and those whose parents did not have university degrees had significantly elevated rates of all barriers. First-year students, heterosexuals, and students at institutions other than HWIs had significantly elevated rates of most barriers.

### Sociodemographic predictors of treatment

Multivariate Poisson regression models for sociodemographic factors and institution type predicting treatment are shown in Table 4 both before and after adjusting for differences in multivariate condition profiles and for perceived need. Age was positively associated with receiving treatment in the total sample and this association was not affected substantially by adjusting for disorder profiles or perceived need. The same general pattern held for being White, beyond the first year of school, having parents who graduated from university, and attending HWIs, each of which was significantly and positively associated with treatment in the total sample as well as without substantial change in this association when adjusting for disorder profile and perceived need.

**Table 4:** Socio-demographic predictors of treatment among students with any diagnosis (multivariable Poisson regression models with dichotomous outcome mediated through types of disorders

	Treatment in the last 12-months for any assessed conditions		Treatment in past 12 months for any condition, controlling for assessed conditions		Treatment in last 12-months for any condition among those with perceived need, controlling for assessed conditions	
	IRR	95% CI	IRR	95% CI	IRR	95% CI
**Age X**^2^(4) p	**42.1**	***p*** **<** 0.001*	**57.5**	***p*** **<** 0.001*	**71.9**	***p*** **<** 0.001*
17–19	–	–	–	–	–	–
20–22	***1.21***	***1.09, 1.35***	***1.16***	***1.05, 1.29***	***1.1***	***1.00, 1.22***
23–25	***1.44***	***1.27, 1.63***	***1.39***	***1.24, 1.56***	***1.3***	***1.16, 1.45***
26–30	***1.37***	***1.19, 1.58***	***1.37***	***1.20, 1.57***	***1.33***	***1.18, 1.51***
31	***1.47***	***1.27, 1.70***	***1.6***	***1.40, 1.84***	***1.66***	***1.46, 1.88***
**Gender** X^2^(2) p	**106.9**	***p*** **<** 0.001*	**31.7**	***p*** **<** 0.001*	**1.8**	***p*** **=** **0.4**
Male	–	–	–	–	–	–
Female	***1.4***	***1.29, 1.52***	***1.21***	***1.12, 1.31***	1.04	0.97, 1.12
Gender non-conforming	***1.76***	***1.41, 2.20***	1.15	0.92, 1.44	1.15	0.95, 1.40
**Population group** X2(2) p	**172.6**	***p*** **<** 0.001*	**114.5**	***p*** **<** 0.001*	**114.1**	***p*** **<** 0.001*
White	–	–	–	–	–	–
Black-other	***0.68***	***0.61, 0.76***	***0.7***	***0.63, 0.77***	***0.72***	***0.66, 0.79***
Black-African	***0.58***	***0.54, 0.62***	***0.64***	***0.60, 0.69***	***0.64***	***0.60, 0.69***
**Student Status** X^2^(1) p	**3.1**	***p*** **=** **0.08**	**0.14**	***p*** **=** **0.70**	**0.18**	***p*** **=** **0.67**
Full-time degree	–	–	–	–	–	–
Part-time degree	0.93	0.84, 1.02	0.98	0.90, 1.08	1.02	0.94, 1.11
**Year in school** X^2^(1) p	**15.6**	***p*** **<** 0.001*	**13.4**	***p*** **<** 0.001*	**6.7**	***p*** **=** 0.009*
1st year	–	–	–	–	–	–
All other	***1.19***	***1.11, 1.28***	***1.18***	***1.10, 1.26***	***1.12***	***1.05, 1.20***
**Parent Education** X^2^(4) p	**28.9**	***p*** **<** 0.001*	**20.8**	***p*** **<** 0.001*	**22.1**	***p*** **<** 0.001*
University Graduate	–	–	–	–	–	–
Less than secondary	***0.83***	***0.74, 0.93***	***0.85***	***0.76, 0.95***	***0.86***	***0.77, 0.95***
Secondary Graduate	***0.83***	***0.76, 0.91***	***0.86***	***0.79, 0.93***	***0.86***	***0.80, 0.93***
Some post-secondary education	***0.87***	***0.79, 0.94***	***0.88***	***0.81, 0.96***	***0.86***	***0.80, 0.93***
**Sexual orientation** X^2^(1) p	**93.5**	***p*** **<** 0.001*	**20.3**	***p*** **<** 0.001*	**8.1**	***p*** **=** 0.004*
Heterosexual	–	–	–	–	–	–
Sexual minority	***1.44***	***1.34, 1.56***	***1.19***	***1.10, 1.28***	***1.11***	***1.04, 1.19***
**Institution type** X^2^(4) p	**126.7**	***p*** **<** 0.001*	**95.3**	***p*** **<** 0.001*	**43.1**	***p*** **<** 0.001*
HWI	–	–	–	–	–	–
DLU	***0.62***	***0.57, 0.69***	***0.67***	***0.61, 0.74***	***0.77***	***0.71, 0.84***
HDI	***0.63***	***0.56, 0.72***	***0.69***	***0.61, 0.77***	***0.78***	***0.70, 0.87***
UT	***0.63***	***0.52, 0.77***	***0.64***	***0.53, 0.77***	***0.71***	***0.59, 0.85***

The term “White” refers to individuals who self-report that they are of European descent, while the term “Black-African” refers to those who self-report that they are of African descent and the term “Black-other” refers to those who self-identify being of ancestry other than European or Black-African

Bold text indiciates a significant association (i.e *p* < 0.05)

Bolditalics text indiciates significant associations

*P < 0/05

The situation was different with gender, as females were significantly more likely than men to receive treatment in the total sample, but this was due entirely to a combination of more severe disorders and greater perceived need. In the case of being a sexual minority, in comparison, there was an elevated rate of treatment in the total sample that was reduced by adjusting for disorder profiles and perceived need, but the association remained significant even after these adjustments. The final predictor, full-time versus part-time student status, was unrelated to probability of receiving treatment.

Table 5 shows the results of an expansion of the multivariable Poisson regression models among those with perceived need after adjusting for disorder profiles, with a focus on respondents that successively excluded those with specific types of barriers. The easiest way to make sense of these results is to focus on χ^2^ values and compare across columns. In doing this it becomes clear that practical barriers are the most important mediators of most significant associations net of disorder profiles and perceived need. Focusing on age, for example, the χ^2^ of 71.9 in the model in the first column of Table 5 is reduced by about 85% of its base value (χ^2^ = 10.0) when we exclude students who reported practical barriers. Comparable proportional χ^2^ reductions associated with practical barriers are 90% for race (χ^2^ changing from 114.1 to 11.7), student year (χ2 changing from 6.7 to 0.3), 87% for parent education (χ^2^ changing from 22.1 to 2.8), and 90% for school type (χ^2^ changing from 43.0 to 4.4). The only exception is sexual orientation, where wanting to handle the problem on their own accounts for a higher proportion of base χ^2^ (89%, from 8.1 to 0.9) than do practical barriers (81%, from 8.1 to 1.5).

**Table 5:** Socio-demographic predictors of treatment (multivariable Poisson regression models with dichotomous outcome among students with perceived need controlling for disorders mediated through barriers

	Treatment for any 12-month condition assessed among students who perceived a need for treatment, controlling for RF		Treatment for any 12-month condition among students who perceived a need for treatment but who did NOT endorse psychological barriers		Treatment for any 12-month condition among students who perceived a need for treatment but who did NOT endorse practical barriers		Treatment for any 12-month condition among students who perceived a need for treatment but who did NOT endorse a preference to talk to family as a barrier to treatment		Treatment for any 12-month condition among students who perceived a need for treatment but who did NOT endorse a preference for handling problems on own as a barrier to treatment		Treatment for any 12-month condition among students who perceived a need for treatment but who did NOT endorse being unsure of the effectiveness of treatment as a barrier to treatment	
	(n = 13,210)		(n = 8486)		(n = 6521)		(n = 10,072)		(n = 7951)		(n = 10,750)	
	IRR	95% CI	IRR	95% CI	IRR	95% CI	IRR	95% CI	IRR	95% CI	IRR	95% CI
**Age X**^2^(4) p	**71.936**	***p*** **<** 0.001*	**25.68**	***p*** **<** 0.001*	**9.964**	***p*** **=** 0.041*	**65.16**	***p*** **<** 0.001*	**20.61**	***p*** **<** 0.001*	**73.78**	***p*** **<** 0.001*
17–19	–	–	–	–	–	–	–	–	–	–	–	–
20–22	***1.1***	***1.00, 1.22***	***1.1***	***1.02, 1.20***	1.05	0.98, 1.13	1.08	0.98, 1.18	1.07	0.99, 1.15	***1.13***	***1.03, 1.23***
23–25	***1.3***	***1.16, 1.45***	***1.23***	***1.12, 1.35***	***1.18***	***1.09, 1.27***	***1.28***	***1.16, 1.42***	***1.11***	***1.01, 1.21***	***1.33***	***1.20, 1.48***
26–30	***1.33***	***1.18, 1.51***	***1.25***	***1.12, 1.38***	***1.12***	***1.03, 1.22***	***1.34***	***1.20, 1.50***	***1.16***	***1.06, 1.28***	***1.4***	***1.25, 1.57***
31	***1.66***	***1.46, 1.88***	***1.37***	***1.23, 1.53***	***1.17***	***1.07, 1.28***	***1.58***	***1.41, 1.78***	***1.31***	***1.19, 1.46***	***1.68***	***1.49, 1.89***
**Gender** X^2^(2) p	**1.834**	***p*** **=** **0.40**	**0.023**	***p*** **=** **0.99**	**1.664**	***p*** **=** **0.44**	**1.468**	***p*** **=** **0.48**	**3.7**	***p*** **=** **0.16**	**0.378**	***p*** **=** **0.83**
Male	–	–	–	–	–	–	–	–	–	–	–	–
Female	1.04	0.97, 1.12	1	0.94, 1.06	1.04	0.99, 1.09	1.04	0.97, 1.11	***0.94***	***0.88, 0.99***	1.02	0.95, 1.09
Other	1.15	0.95, 1.40	0.99	0.84, 1.16	***1.11***	***1.01, 1.22***	1.09	0.91, 1.31	0.95	0.82, 1.11	1.05	0.88, 1.26
**Population group** X2(2) p	**114.12**	***p*** **<** 0.001*	**39.4**	***p*** **<** 0.001*	**11.74**	***p*** **=** 0.003*	**70.61**	***p*** **<** 0.001*	**38.05**	***p*** **<** 0.001*	**57.73**	***p*** **<** 0.001*
White	–	–	–	–	–	–	–	–	–	–	–	–
Black-other	***0.72***	***0.66, 0.79***	***0.82***	***0.76, 0.88***	***0.93***	***0.88, 0.98***	***0.77***	***0.71, 0.84***	***0.86***	***0.81, 0.92***	***0.76***	***0.70, 0.83***
Black-African	***0.64***	***0.60, 0.69***	***0.77***	***0.74, 0.82***	***0.87***	***0.84, 0.91***	***0.71***	***0.67, 0.75***	***0.78***	***0.74, 0.82***	***0.74***	***0.69, 0.78***
**Student Status** X^2^(1) p	**0.184**	***p*** **=** **0.67**	**0.004**	***p*** **=** **0.95**	**0.032**	***p*** **=** **0.86**	**0.39**	***p*** **=** **0.53**	**0.014**	***p*** **=** **0.91**	**0.044**	***p*** **=** **0.84**
Full-time degree	–	–	–	–	–	–	–	–	–	–	–	–
Part-time degree	1.02	0.94, 1.11	1	0.93, 1.07	1.01	0.95, 1.07	1.03	0.95, 1.11	1	0.94, 1.08	1.01	0.93, 1.09
**Year in school** X^2^(1) p	**6.748**	***p*** **=** 0.009*	**1.236**	***p*** **=** **0.21**	**0.984**	***p*** **=** **0.32**	**8.04**	***p*** **=** 0.005*	**3.328**	***p*** **=** **0.07**	**3.967**	***p*** **=** 0.046*
1st year	–	–	–	–	–	–	–	–	–	–	–	–
All other	***1.12***	***1.05, 1.20***	***1.05***	***1.00, 1.11***	***1.05***	***1.00, 1.10***	***1.13***	***1.07, 1.20***	***1.09***	***1.03, 1.14***	***1.09***	***1.03, 1.16***
**Parent Education** X^2^(4) p	**22.141**	***p*** **<** 0.001*	**6.467**	***p*** **=** **0.09**	**2.769**	***p*** **=** **0.43**	**12.75**	***p*** **=** 0.005*	**8.327**	***p*** **=** 0.04*	**9.94**	***p*** **=** 0.019*
University Graduate	–	–	–	–	–	–	–	–	–	–	–	–
Less than secondary	***0.86***	***0.77, 0.95***	1.02	0.94, 1.11	1.01	0.94, 1.08	***0.89***	***0.81, 0.98***	0.89	0.81, 0.96	0.93	0.84, 1.02
Secondary Graduate	***0.86***	***0.80, 0.93***	0.98	0.91, 1.04	***1.05***	***1.00, 1.10***	***0.91***	***0.85, 0.98***	***0.93***	***0.87, 0.99***	***0.91***	***0.85, 0.98***
Some post-secondary education	***0.86***	***0.80, 0.93***	***0.91***	***0.86, 0.97***	0.97	0.93, 1.03	***0.88***	***0.82, 0.94***	***0.92***	***0.86, 0.97***	***0.89***	***0.83, 0.96***
**Sexual orientation** X^2^(1) p	**8.118**	***p*** **=** ***0.004***	**4.187**	***p*** **=** ***0.041***	**1.513**	***p*** **=** **0.22**	**4.803**	***p*** **=** **0.03**	**0.909**	***p*** **=** **0.34**	**7.164**	***p*** **=** ***0.007***
Heterosexual	–	–	–	–	–	–	–	–	–	–	–	–
Sexual minority	***1.11***	***1.04, 1.19***	***1.08***	***1.02, 1.14***	***1.05***	***1.00, 1.10***	***1.09***	***1.02, 1.16***	1.04	0.98, 1.09	***1.11***	***1.04, 1.18***
**Institution type** X^2^(4) p	**43.053**	***p*** **<** 0.001*	**13.96**	***p*** **=** 0.003*	**4.426**	**p** **=** **0.22**	**34.18**	***p*** **=** 0.028*	**11.44**	***p*** **=** 0.01*	**35.24**	***p*** **<** 0.001*
HWI	–	–	–	–	–	–	–	–	–	–	–	–
DLU	***0.77***	***0.71, 0.84***	***0.87***	***0.81, 0.94***	0.97	0.92, 1.03	***0.8***	***0.74, 0.86***	***0.87***	***0.82, 0.93***	***0.79***	***0.73, 0.85***
HDI	***0.78***	***0.70, 0.87***	***0.84***	***0.76, 0.92***	***0.92***	***0.85, 0.99***	***0.8***	***0.72, 0.89***	***0.87***	***0.79, 0.95***	***0.81***	***0.73, 0.90***
UT	***0.71***	***0.59, 0.85***	***0.84***	***0.72, 0.98***	***0.83***	***0.73, 0.95***	***0.72***	***0.60, 0.85***	0.87	0.75, 1.01	***0.72***	***0.60, 0.85***

The term “White” refers to individuals who self-report that they are of European descent, while the term “Black-African” refers to those who self-report that they are of African descent and the term “Black-other” refers to those who self-identify being of ancestry other than European or Black-African

Bold text indiciates a significant association (i.e *p* < 0.05)

Bolditalics text indiciates significant associations

*P < 0/05

## Discussion

Our survey found high rates of mental health problems and self-harm, low treatment rates, and many barriers reported by students who did not get treatment. Somewhat less than two-thirds (60.5%) of students with any of the problems assessed perceived themselves as needing treatment, although the rate of perceived need was higher among individuals reporting self-harm (72.1%), and higher among those reporting severe symptoms of any disorder and/or self-harm (82.3%). These results highlight the large unmet need for mental health treatment on SA university campuses consistent with previous studies showing similar patterns internationally [1, 2, 35], and in a prior SA study [3, 4]. Given the large number of students in need of treatment, traditional models of psychological intervention relying on one-to-one psychotherapy will not be a feasible or sustainable response. Innovative sustainable solutions, including the use of emerging technology (such as smartphone applications), peer-to-peer support, and group interventions could be part of the solution. It will be important to develop these novel interventions in consultation with students to ensure that they are student-centered, acceptable, and accessible, especially considering our finding that students’ utilization of mental healthcare is impeded by psychological, practical, and other barriers to treatment seeking. Designing services that explicitly take account of the barriers reported by students is integral to closing the mental health treatment gap on SA university campuses.

Importantly, the considerably lower treatment gap in HWIs compared to other institutions was explained by differences in practical barriers, suggesting that the greater resources available in HWIs are important facilitators of access to treatment and that increasing resources at HDIs could be integral to addressing the treatment gap. We also observed significant sociodemographic differences in access to treatment, again due more to practical barriers than to perceived need or other barriers. These data suggest that targeted interventions to engage vulnerable segments of the student population could be important in redressing inequalities in differential access to mental health services on university campuses in SA [16].

It is noteworthy that only 60.5% (S.E. = 0.5) of students with mental health problems reported that they perceived a need for treatment given that understanding and accepting this need is a prerequisite for accessing treatment. Indeed, some students maybe correct that formal treatment (which would position them as mental health service users) is not what is needed for their particular problems, particularly in the context of social problems such as violence and economic inequalities which may precipitate psychological distress but which do not require psychological treatments. Nonetheless, improving students’ mental health literacy (i.e., knowledge and attitudes required to recognize, manage, and prevent mental disorders, as well as appropriate help-seeking behaviors) is one way to help students recognize when they need psychological interventions and increase recognition that treatment can be helpful [36]. Previous research has shown that mental health literacy is associated with good student mental health []. Mental health literacy training programmes for students have been developed and implemented with promising results [36, 37]. Broader psycho-educational interventions have also demonstrated success at reducing stigma and myths about mental health [36].

Practical barriers (including concerns about costs, not knowing how to access treatment and scheduling difficulties) were not only most important in accounting for sociodemographic correlates of treatment but also the single most common type of barrier in our study. This has important implications for planning services. As noted, digital interventions, including smartphone applications and chatbots could be one way to increase accessibility and convenience as well as reduce costs [38]. A review of students’ experiences with and attitudes toward such technology-assisted interventions concluded that students view them as convenient, accessible, easy to use, and helpful, as well as overcoming the barrier of stigma associated with seeking treatment [39]. There is also growing evidence that digital mental health Interventions for anxiety, depression, and enhanced well-being are effective among university students [40]. Preliminary studies suggest that results might be similar among SA students [41, 42], although more research is required. While digital interventions may be more cost-effective than traditional therapies they are not without any costs; problems associated with unequal access to technology and internet access in SA could exacerbate inequality in access to treatment particularly among the most economically vulnerable students if digital interventions are widely implemented.

Finally, it is unsurprising that students report preferences to talk to family/friends and use self-reliance as reasons for not accessing treatment, given that most undergraduate students are young adults and that young adulthood is a developmental period marked by striving for autonomy, self-reliance and (appropriate) distrust of authority and tradition. This highlights the importance of ensuring that new services are delivered in ways that support the developmental trajectory of young adults, including providing opportunities for autonomy, self-reliance, and peer-support [18].

When interpreting the findings of this study it is important to note that risk ratios (RR) observed for significant associations are for the most part modest. These modest RRs are only significant by virtue of the large sample size which has allowed us to estimate RRs accurately with narrow confidence intervals. Care thus needs to be exercised not to over-interpret observed associations which are significant but nonetheless have small RRs.

This study has several limitations including the use of nonprobability sampling, a reliance on self-report measures, and the fact that 9 universities in the country did not participate in the study. Our reliance on a convenience sample together with the relatively low and quite variable response rates across institutions may limit the generalizability of results, although we corrected for this to the extent possible by weighting the data. Nonetheless, this study is the first of its kind to systematically investigate mental healthcare utilisation and barriers to treatment seeking among students from many SA universities and provide insights into the need for interventions and strategies to reduce the mental health treatment gap among the country’s students. Furthermore, the methodology we have used to analyse these data is novel, even by international standards.

## Conclusion

Mental health problems are highly prevalent among SA university students but are seldom treated. Disparities in treatment rates are observed across the various kinds of institutions and different sociodemographic groups, with reduced probability of obtaining treatment (net of condition profiles) associated with young age, genders other than female, and a range of indicators of social disadvantage (first year of study, atypical sexual orientation, part-time student status, low parent education, and attending institutions other than HWIs). A lack of perceived need for treatment is partially responsible for the low treatment rates, but more important are a range of practical and psychological/attitudinal barriers, a preference to talk to family/friends, a preference for self-reliance, and doubts about the effectiveness of treatments. Crucially, practical barriers seem to be especially important in accounting for the associations observed between not accessing treatment and indicators of social disadvantage.

### Supplementary Information

**Additional file 1**. Supplementary Tables.

## Data Availability

Availability of data and datasets used and/or analysed during the current study are available from the corresponding author on reasonable request.

## References

[1] Duffy A, Saunders KEA, Malhi GS, Patten S, Cipriani A, McNevin SH (2019). Mental health care for university students: a way forward?. Lancet Psychiatry.

[2] Storrie K, Ahern K, Tuckett A (2010). A systematic review: students with mental health problems—a growing problem. Int J Nurs Pract.

[3] Bantjes J, Lochner C, Saal W, Roos J, Taljaard L, Page D (2019). Prevalence and sociodemographic correlates of common mental disorders among first-year university students in post-apartheid South Africa: implications for a public mental health approach to student wellness. BMC Public Health.

[4] Bantjes J, Kessler M, Lochner C, Breet E, Bawa A, Roos J (2023). The mental health of university students in South Africa: results of the national student survey. J Affect Disord.

[5] Bantjes J, Kessler MJ, Hunt X, Kessler RC, Stein DJ (2023). Prevalence and correlates of 30-day suicidal ideation and intent: results of the South African National Student Mental Health Survey. SAMJ S Afr Med J.

[6] Auerbach RP, Mortier P, Bruffaerts R, Alonso J, Benjet C, Cuijpers P (2018). WHO World Mental Health Surveys International College Student Project: prevalence and distribution of mental disorders. J Abnorm Psychol.

[7] Mortier P, Auerbach RP, Alonso J, Bantjes J, Benjet C, Cuijpers P (2018). Suicidal thoughts and behaviors among first-year college students: results from the WMH-ICS project. J Am Acad Child Adolesc Psychiatry.

[8] Bantjes J, Kessler MJ, Hunt X, Kessler RC, Stein DJ (2023). Prevalence and correlates of 30-day suicidal ideation and intent: results of the South African National Student Mental Health Survey. S Afr Med J.

[9] Alonso J, Vilagut G, Mortier P, Auerbach RP, Bruffaerts R, Cuijpers P (2019). The role impairment associated with mental disorder risk profiles in the WHO World Mental Health International College Student Initiative. Int J Methods Psychiatr Res.

[10] Bantjes J, Saal W, Gericke F, Lochner C, Roos J, Auerbach RP (2020). Mental health and academic failure among first-year university students in South Africa. S Afr J Psychol.

[11] Kiekens G, Claes L, Demyttenaere K, Auerbach RP, Green JG, Kessler RC (2016). Lifetime and 12-month nonsuicidal self-injury and academic performance in college freshmen. Suicide Life Threat Behav.

[12] Haas AP, Hendin H, Mann JJ (2016). Suicide in college students. Am Behav Sci.

[13] Cuijpers P, Miguel C, Ciharova M, Aalten P, Batelaan N, Salemink E (2021). Prevention and treatment of mental health and psychosocial problems in college students: an umbrella review of meta-analyses. Clin Psychol Sci Pract.

[14] Hunt J, Eisenberg D (2010). Mental health problems and help-seeking behavior among college students. J Adolesc Health.

[15] Osborn TG, Li S, Saunders R, Fonagy P (2022). University students’ use of mental health services: a systematic review and meta-analysis. Int J Ment Health Syst.

[16] Bantjes J, Saal W, Lochner C, Roos J, Auerbach RP, Mortier P (2020). Inequality and mental healthcare utilisation among first-year university students in South Africa. Int J Ment Health Syst.

[17] Lui JC, Sagar-Ouriaghli I, Brown JSL (2022). Barriers and facilitators to help-seeking for common mental disorders among university students: a systematic review. J Am Coll Health.

[18] Bantjes J, Hunt X, Stein DJ (2022). Public health approaches to promoting university students’ mental health: a global perspective. Curr Psychiatry Rep.

[19] Nattrass N, Nattrass J (2008). South Africa, the homelands and rural development. Dev South Afr.

[20] Council on Higher Education. Universities of Technology-Deepening the Debate. Pretoria; 2010. www.jacana.co.za

[21] Cuijpers P, Auerbach RP, Benjet C, Bruffaerts R, Ebert D, Karyotaki E (2019). Introduction to the special issue: The WHO World Mental Health International College Student (WMH-ICS) initiative. Int J Methods Psychiatr Res.

[22] Kessler RC, Calabrese JR, Farley PA, Gruber MJ, Jewell MA, Katon W (2013). Composite International Diagnostic Interview screening scales for DSM-IV anxiety and mood disorders. Psychol Med.

[23] Kessler RC, Santiago PN, Colpe LJ, Dempsey CL, First MB, Heeringa SG (2013). Clinical reappraisal of the Composite International Diagnostic Interview Screening Scales (CIDI-SC) in the Army Study to Assess Risk and Resilience in Servicemembers (Army STARRS). Int J Methods Psychiatr Res.

[24] Babor TF, Higgins-Biddle J, Dauser D, Higgins P, Burleson JA (2005). Alcohol screening and brief intervention in primary care settings: implementation models and predictors. J Stud Alcohol.

[25] Reinert DF, Allen JP (2002). The alcohol use disorders identification test (AUDIT): a review of recent research. Alcohol Clin Exp Res.

[26] Posner K, Brown GK, Stanley B, Brent DA, Yershova KV, Oquendo MA (2011). The Columbia-suicide severity rating scale: Initial validity and internal consistency findings from three multisite studies with adolescents and adults. Am J Psychiatry.

[27] Kazis LE, Selim AJ, Rogers W, Qian SX, Brazier J (2012). Monitoring outcomes for the medicare advantage program: methods and application of the VR-12 for evaluation of plans. J Ambul Care Manag.

[28] Jones D, Kazis L, Lee A, Rogers W, Skinner K, Cassar L (2001). Health status assessments using the Veterans SF-12 and SF-36: methods for evaluating otucomes in the Veterans Health Administration. J Ambul Care Manag.

[29] Groves RM (2012). Nonresponse in household interview surveys.

[30] van Buuren S (2007). Multiple imputation of discrete and continuous data by fully conditional specification. Stat Methods Med Res.

[31] Rubin DB (1987). Multiple imputation for nonresponse in surveys.

[32] Wolter KM. Taylor series methods. In: Introduction to variance estimation. 2007;226–71. 10.1007/978-0-387-35099-8_6

[33] Breiman L (2001). Random forests. Mach Learn.

[34] Chen W, Qian L, Shi J, Franklin M (2018). Comparing performance between log-binomial and robust Poisson regression models for estimating risk ratios under model misspecification. BMC Med Res Methodol.

[35] Bruffaerts R, Mortier P, Auerbach RP, Alonso J, Hermosillo De la Torre AE, Cuijpers P (2019). Lifetime and 12-month treatment for mental disorders and suicidal thoughts and behaviors among first year college students. Int J Methods Psychiatr Res.

[36] Reis AC, Saheb R, Moyo T, Smith C, Sperandei S (2022). The impact of mental health literacy training programs on the mental health literacy of university students: a systematic review. Prev Sci.

[37] Tay JL, Tay YF, Klainin-Yobas P (2018). Effectiveness of information and communication technologies interventions to increase mental health literacy: a systematic review. Early Interv Psychiatry.

[38] Lattie EG, Lipson SK, Eisenberg D (2019). Technology and college student mental health: Challenges and opportunities. Front Psychiatry.

[39] Hadler NL, Bu P, Winkler A, Alexander AW (2021). College student perspectives of telemental health: a review of the recent literature. Curr Psychiatry Rep.

[40] Lattie EG, Adkins EC, Winquist N, Stiles-Shields C, Wafford QE, Graham AK (2019). Digital mental health interventions for depression, anxiety, and enhancement of psychological well-being among college students: systematic review. J Med Internet Res.

[41] Gericke F, Ebert DD, Breet E, Auerbach RP, Bantjes J (2021). A qualitative study of university students’ experience of Internet-based CBT for depression. Couns Psychother Res.

[42] Gericke F, Ebert DD, Breet E, Auerbach RP, Bantjes J (2021). A qualitative study of university students’ experience of Internet-based CBT for depression. Couns Psychother Res.

