# Supplementary material for: Treatment rates and barriers to mental health service utilisation among university students in South Africa
Source: Int J Ment Health Syst. 2023 Nov 9;17:38. doi: 10.1186/s13033-023-00605-7 (PMC10633973; doi:10.1186/s13033-023-00605-7)
Supplement: Supplementary file 1 — Additional file 1. Supplementary Tables. [file 13033_2023_605_MOESM1_ESM.docx]

**Supplementary Tables**

| **Table S1.** Participating institutions, population sizes, sample sizes, and sample weights | | | | | |
| --- | --- | --- | --- | --- | --- |
|  |  |  |  |  |  |
|  | **Population size** |  | **Sample size** |  | **Sample** |
| I. Historically White Institutions | **%** |  | **%** |  | **Weight** |
| University of Cape Town | 2.6 |  | 3.6 |  | 0.7 |
| University of the Free State | 5.3 |  | 4.3 |  | 1.2 |
| University of Johannesburg | 5.9 |  | 3.6 |  | 1.6 |
| Nelson Mandela University | 3.9 |  | 1.7 |  | 2.3 |
| University of Pretoria | 0.6 |  | 0.3 |  | 1.9 |
| Rhodes University | 0.9 |  | 2.3 |  | 0.4 |
| Stellenbosch University | 3.1 |  | 4.4 |  | 0.7 |
| University of the Witwatersrand | 3.8 |  | 1.9 |  | 2.0 |
| Total | 26.1 |  | 22.1 |  |  |
| (n) | (171,517) |  | (6,238) |  |  |
| II. Historically Disadvantaged Institutions | |  |  |  |  |
| University of Limpopo | 3.3 |  | 2.2 |  | 1.5 |
| Sefako Makgatho Health Sciences University | 0.9 |  | 0.5 |  | 1.8 |
| Walter Sisulu University of Technology & Science | 4.1 |  | 3.1 |  | 1.3 |
| University of the Western Cape | 3.6 |  | 3.0 |  | 1.2 |
| Total | 11.9 |  | 8.8 |  |  |
| (n) | (78,374) |  | (2,497) |  |  |
| III. Universities of Technology | |  |  |  |  |
| Durban University of Technology | 4.8 |  | 1.4 |  | 3.5 |
| Mangosuthu University of Technology | 2.1 |  | 0.3 |  | 7.6 |
| University of Mpumalanga | 0.6 |  | 0.3 |  | 2.1 |
| Sol Plaatje University | 0.4 |  | 0.7 |  | 0.7 |
| Total | 7.9 |  | 2.7 |  |  |
| (n) | (52,486) |  | (750) |  |  |
| IV. Distance Learning University | |  |  |  |  |
|  | 54.0 |  | 66.4 |  | 0.8 |
| (n) | (355,055) |  | (18,783) |  |  |
| V. Total |  |  |  |  |  |
| (n) | (657,432) |  | (28,268) |  |  |
|  | | | | | |

**Table S2.** Standardized partial regression coefficients from three-factor principal axis factor analysis with promax rotation

**I II III**

**I. Psychological**

Too embarrassed 0.54 0.07 0.10 Afraid it might harm school or professional career 0.51 0.24 0.00

Worried people would treat you differently 1.0 -0.11 -0.17

**II. Practical**

Unsure where to go 0.02 0.70 -060

Too expensive -0.07 0.66 -0.03

Problems with time, transportation, scheduling 0.06 0.63 -0.04

**III. Other**

Want to handle on own 0.19 -0.26 0.77

Talk to friends/relatives instead -0.08 0.05 0.24

Not sure available treatments are very effective 0.13 0.29 0.13

**Table S2:** Distribution of students in the Venn diagram

| **Unsure of effectiveness** | **Prefer to handle it on their own** | **Prefer to talk to family / friends** | **Psychological barriers** | **Practical barriers** | **Total** | **Proportion (%)** |
| --- | --- | --- | --- | --- | --- | --- |
| + | - | - | - | - | 35 | 0.4 |
| - | + | - | - | - | 321 | 3.8 |
| - | - | + | - | - | 167 | 2 |
| - | - | - | + | - | 83 | 1 |
| - | - | - | - | + | 667 | 8 |
| + | - | - | - | + | 198 | 2.4 |
| + | - | - | + | + | 278 | 3.3 |
| + | - | - | + | - | 14 | 0.2 |
| - | - | - | + | + | 538 | 6.4 |
| - | - | + | + | + | 207 | 2.5 |
| + | - | + | + | + | 135 | 1.6 |
| + | + | - | + | - | 56 | 0.7 |
| + | + | - | + | + | 624 | 7.4 |
| + | + | + | + | + | 487 | 5.8 |
| + | + | + | + | - | 25 | 0.3 |
| + | - | + | + | - | 13 | 0.2 |
| - | - | + | + | - | 40 | 0.5 |
| - | - | + | - | + | 309 | 3.7 |
| + | - | + | - | + | 78 | 0.9 |
| + | + | - | - | + | 170 | 2 |
| - | + | + | + | + | 573 | 6.8 |
| - | + | - | + | + | 999 | 11.9 |
| + | + | + | - | + | 120 | 1.4 |
| - | + | + | - | + | 419 | 5 |
| + | - | + | - | - | 53 | 0.6 |
| + | + | + | - | - | 36 | 0.4 |
| + | + | - | - | - | 53 | 0.6 |
| - | + | + | - | - | 307 | 3.7 |
| - | + | + | + | - | 102 | 1.2 |
| - | + | - | - | + | 481 | 5.7 |
| - | + | - | + | - | 229 | 2.7 |
